# London Hospitals with Medical Schools

**Published:** 1919-12-20

**Authors:** 


### THE THREE LEAN HOSPITAL YEARS AHEAD.

### REPORTS FROM CHAIRMEN THROUGHOUT THE COUNTRY.

[The splendid readiness of Hospital Chairmen to co-operate in making the voluntary hospitals secure for the future is eloquently testified by the information and answers which we have received in less than a week from the day we approached them. We extend to them our grateful thanks with the assurance that they may depend upon the continuous co-operation of "The Hospital" and its staff until complete victory for the voluntary hospitals is secured.]

### LONDON HOSPITALS WITH MEDICAL SCHOOLS.

### GUY'S HOSPITAL, London Bridge, S.E. 1.

(*a*) Immediate Needs.

£20,000 additional subscriptions and donations to meet the great rise in expenditure.

(*b*) Needs for 1920.

The Charity is dependent upon support from sources other than its endowments to the extent of over £60,000 per annum.

(*Signed*) F. P. WHITBREAD, *Chairman.*

### KING'S COLLEGE HOSPITAL.

(*a*) Immediate Needs.

£50,000 to pay off debt.

(*b*) Needs for 1920.

£60,000, maintenance and administration; £50,000, additions to Nurses' Home (increased staff required for forty-eight hours' week); £15,000, development of physio-therapeutic department.

(*Signed*) HAMBLEDEN [Viscount], *Chairman.*

Continued on page 270.

### LONDON HOSPITALS: CHAIRMEN'S REPORTS—(*continued.*)

### LONDON HOSPITAL.

(*a*) Immediate Needs.

Unless some wonderful change takes place, will owe its bankers at least £50,000 at end of year 1919. Must build a long-delayed hostel for residents, and pay balance (£10,000) yet unpaid on Cavell Home for Nurses.

(*b*) Needs for 1920.

Increased subscriptions for upkeep, in addition to above capital expenditure. The capital expenditure is not wanted with any view of increasing the work, but merely to do the present work with greater efficiency.

(*Signed*) KNUTSFORD [Viscount], *Chairman.*

### ROYAL FREE HOSPITAL, Gray's Inn Road, W.C.

(*a*) Immediate Needs.

£25,000 to repay loan to bank. £20,000, approximate deficit in income during 1919.

(*b*) Needs for 1920.

£20,000 to meet the estimated deficit during 1920.

(*Signed*) ALFRED LANGTON, *Chairman.*

### ST. BARTHOLOMEW'S HOSPITAL.

(*a*) Immediate Needs.

Funds for the Nurses' Home rebuilding, £150,000.

(*b*) Needs for 1920.

£40,000 a year for maintenance purposes.

(*Signed*) SANDHURST [Viscount], Chairman.

### ST. GEORGE'S HOSPITAL.

(*a*) Immediate Needs.

£7,000 for current expenditure. £500 for additional apparatus and alterations in electro-therapeutic department.

(*b*) Needs for 1920.

£500 for new furniture for nurses' quarters. £20,000 for current expenditure.

(*Signed*) F. J. FRANKAU, *Chairman.*

### WESTMINSTER HOSPITAL.

(*a*) Immediate Needs.

£15,000 to meet the present deficit.

(*b*) Needs for 1920.

£20,000 additional income.

(*Signed*) GLENCONNER [Lord], *Chairman.*

